# Shear Strength of Lightweight Concrete Structural Elements Reinforced with FRP Bars: Experimental Studies vs. Code Predictions

**DOI:** 10.3390/ma18153525

**Published:** 2025-07-27

**Authors:** Agnieszka Wiater, Tomasz Wojciech Siwowski

**Affiliations:** Department of Roads and Bridges, The Faculty of Civil and Environmental Engineering and Architecture, Rzeszow University of Technology, 35-959 Rzeszów, Poland; wiater@prz.edu.pl

**Keywords:** lightweight concrete, FRP reinforcement, beam, slab, shear strength, experiment, code prediction

## Abstract

Using lightweight concrete (LWC) reduces the dead weight of the concrete structure by 25–30% compared to ordinary concrete. However, harmful and corrosive substances penetrate the lightweight concrete matrix due to its high permeability, resulting in higher maintenance costs and a reduced structure service life. Therefore, in harsh environments where conventional steel bars are susceptible to corrosion, fibre-reinforced polymer (FRP) bars should be used for reinforcement. However, there is a paucity of experimental studies regarding LWC structural elements reinforced with FRP bars. Shear strength is a critical limit state that typically determines the proper design of such elements, ensuring the required safety margin and an appropriate level of reliability. The research work was conducted to compare the experimentally determined shear strengths (V_exp_) of 50 structural elements (beams, slabs) made of LWC/FRP with code predictions (V_code_) made according to eight codes used for design. Based on this comparison, the so-called conformity coefficient (V_exp_/V_code_) was calculated and used to assess which provision documents are the best, considering the entire population of test results. The work demonstrated that the recent Eurocode best predicts the shear strength of LWC/FRP elements.

## 1. Introduction

Lightweight concrete (LWC) has a bulk density in the dry state of not more than 2000 kg/m^3^ and a compressive strength of up to 60 MPa. Its use reduces the dead weight of the concrete structure by 25–30% compared to ordinary concrete. Moreover, the dimensions of the structural elements can be significantly reduced, thereby decreasing construction costs. Compared to ordinary concrete, lightweight concrete exhibits greater durability, enhanced resistance to freezing–thawing cycles (due to the higher porosity of lightweight aggregate), and improved fire resistance, as well as significantly enhanced seismic resistance of the structure [1,2,3]. In the case of modernised structures, using LWC, it is also possible to increase the load-bearing capacity of the existing structure, reducing its dead weight. LWC has 25–50% lower tensile strength and modulus of elasticity compared to ordinary concrete, while its creep rates are comparable. In civil engineering, lightweight concrete has numerous applications, such as skyscrapers, large-span bridges, and ocean platforms [4,5,6,7,8].

Lightweight aggregates of mineral origin are used in the production of lightweight concrete. Depending on the material from which they are made, mineral aggregates are divided into rock aggregates, obtained from natural rocks and artificial aggregates, made from mineral raw materials and industrial waste as a result of heat treatment, or obtained from raw materials of organic origin. The increasing use of lightweight artificial aggregates is due to the limited stock of rock aggregates and the need to dispose of industrial waste. It is imperative in the sustainable development policy recommended by the European Union and other highly developed countries [9,10,11]. A characteristic feature common to all lightweight aggregates is their porous structure; as a rule, their strength is less than that of hardened cement grout. As a result, concrete made from these aggregates differs from ordinary concrete in terms of bulk density and other properties, as well as in the technology used to produce it.

The production of lightweight concrete is continually evolving, and research is being conducted to enhance the material properties of LWC. As a result, lightweight aggregate/high-performance concrete (LWA/HPC) was developed, a natural direction for creating lightweight structural concrete [2,5,12]. The LWA/HPC has been primarily used in elements of ocean platforms to ensure floating during the initial construction phase, as well as in bridges and roofs with large spans. Currently, the compressive strength of these concretes is achieved in the range of LC60 to LC85 with a density of 1850 to 2000 kg/m^3^. They are dominated by the use of artificial aggregates from sintered fly ash (lytag), swollen clays (liapor, leca), or swelling shale (solite, hydite). Other components are characteristic materials for high-quality concretes, including plasticisers and superplasticisers, as well as mineral additives such as silica dust and fly ash [13]. Therefore, one can hope that the global achievements in developing lightweight concrete will stimulate the research, design, and implementation works in Poland and other Eastern European countries in civil engineering. It can be essential in bridge construction, where reducing the dead weight of the structure by up to 25% [14] is not insignificant. Although aggregates for lightweight concrete are currently more expensive than natural aggregates, this solution may prove more advantageous in life cycle calculations. Domestic deposits of natural aggregates are significant because they are being depleted, and the possibility of producing artificial aggregates, especially from post-industrial waste, remains underutilised.

Harmful and corrosive substances can penetrate the lightweight concrete matrix due to its high permeability. Structures made from lightweight concrete (LWC), such as bridges, parking garages, water tanks, and offshore platforms, are often exposed to harsh environmental conditions. This exposure can lead to the corrosion of steel reinforcement and the eventual deterioration of the concrete, resulting in higher maintenance costs and a reduced service life for the structure. In environments where conventional steel bars are prone to corrosion, fibre-reinforced polymer (FRP) bars should be used instead for reinforcement. FRP composites offer several advantages, including corrosion resistance, high tensile strength, a favourable strength-to-weight ratio, electrical non-conductivity, and lightweight properties—typically about one-quarter the weight of steel bars. Additionally, FRP bars are easy to install and adaptable, making them an attractive option for enhancing the durability of LWC structures. As a result, FRP bars are increasingly being accepted in many design codes as an alternative reinforcement to traditional steel bars. However, it is important to keep in mind that while using FRP as reinforcement in reinforced concrete (RC) elements, it has some limitations. FRP has a modulus of elasticity that is about one-third that of steel bars, and while it possesses high tensile strength, it lacks a yielding point. Furthermore, FRP shows considerably lower tensile strength in bent portions compared to straight portions and has lower dowel resistance.

Combining different materials and technologies to improve performance and increase the effectiveness of structures is one of the research and development paths in civil engineering. However, there is a paucity of experimental studies regarding LWC elements (beams, slabs) reinforced with FRP bars (hereinafter referred to as LWC/FRP) [15,16,17,18]. The vast majority of standard procedures for designing concrete elements with FRP reinforcement concern elements made of ordinary concrete. According to several preliminary studies [16,19], when using LWC, these procedures are not sufficiently accurate, safe, or optimal regarding material consumption. It can lead to incorrect dimensioning of the designed elements, resulting in exceeding the limit states during the operation of structures and/or increased consumption of expensive materials. None of the procedures used in modern standards for the design of concrete elements reinforced with FRP bars allows for checking all limit states required in the design of LWC structures with satisfactory accuracy. Based on the review and preliminary analysis of the literature, the authors determined that the critical conditions determining the possibility of using LWC/FRP elements, e.g., in bridge decks, are shear strength and fatigue life. The fatigue life of LWC/FRP elements has been the subject of previous research by the authors [20]. This paper presents the results of research and analysis on the shear strength of LWC/FRP elements. Shear strength is a critical limit state that typically determines the proper design of such elements, ensuring the required safety margin and an appropriate level of reliability. This limit state has been analysed in detail in this paper to develop the best calculation procedure. This is a continuation of the author’s work, as published in papers [14,19], but with a significantly larger database of their own and experimental results.

Despite extensive research conducted over the last decade, accurately determining the shear strength of concrete structures reinforced with FRP bars remains a challenging task [21,22,23,24]. Research has shown that FRP composites significantly delay the growth of critical diagonal cracks and substantially improve the load-carrying capacity of concrete elements [25]. However, concrete elements reinforced with FRP bars without transverse reinforcement (stirrups) show lower shear strength than those reinforced with steel bars [26]. On the other hand, the shear strength of lightweight concrete is less than that of ordinary concrete [27]. Previous studies and comparisons in the field of LWC/FRP have included a maximum of 18 elements [16,28]. Additionally, half of the data in this database is 10 years old and does not account for current provision documents. Therefore, the conclusions are partly outdated because they do not account for subsequent tests and new standards.

This study used research to compare the shear strengths of LWC/FRP elements (beams and slabs) determined experimentally by various researchers (and authors) versus code predictions made according to several standards or guidelines used or recommended for design. In reviewing the literature on experimental studies, the types of elements, LWC, and FRP composite bars were addressed, and the shear strength was investigated using data published in subsequent papers. A total of 50 tested elements made of LWC/FRP were considered in this study. The collected experimental test results were then compared with the relevant calculated values determined according to the empirical equations proposed in eight provision documents, which contain a procedure for calculating the shear strength of concrete elements reinforced with FRP bars without stirrups [29,30,31,32,33,34,35,36]. Based on this comparison, the so-called conformity coefficient (V_exp_/V_code_) was determined and used to assess which standard procedure is the best, considering the entire population of test results. It was also determined whether it is worthwhile to standardise one procedure for the entire population or whether it is better to adopt separate procedures for different LWCs and FRP bars to achieve better compliance with test results. The directions of potential changes are also given in selected code procedures to obtain better conformity coefficient values (i.e., closer to 1.0).

## 2. Review of Experimental Works

Until now, experimental tests of LWC/FRP structural elements have rarely been performed. Between 2013 and 2023, to the best of the authors’ knowledge, only six research papers were published that presented research results on lightweight concrete beams or slabs reinforced with FRP composite bars. The scope of these investigations, along with the most important conclusions drawn from them, is briefly presented below. Since standard procedures do not distinguish between flexural elements without transverse reinforcement for beams and slabs, the review considered the test results for both types of structural elements together. In addition, some of the results of the discussed research and the conclusions drawn from them are related to design procedures based on outdated standards, e.g., [37,38,39]. A corresponding comment has been added to the description.

Liu and Pantelides [16] conducted a research program to investigate the shear behaviour of lightweight concrete (LWC) and glass fibre-reinforced polymer (GFRP) slabs, comparing the test results with predictions from three different design guidelines. They tested twelve LWC slabs using a three-point bending scheme. The variables in the study included concrete compressive strength, reinforcement ratio, slab thickness, deck span, and slab width. Based on their research, they established a conservative reduction factor of λf = 0.80 for the ACI design guide [37], which has since been replaced by the current ACI code [36]. This reduction factor allows the modified shear strength equation for LWC slabs to exhibit a similar level of conservatism to that of normal-weight concrete (NWC) slabs. The Canadian standard [39], which has also been updated by standard [33], and the Japanese recommendation [29] predict the shear strength of LWC/GFRP slabs with the same degree of conservatism as achieved with NWC slabs, eliminating the need for a reduction factor in these guidelines. However, the authors concluded that additional tests on LWC/GFRP slabs are necessary to establish a more appropriate reduction factor that is less conservative.

Kim and Jang [28] proposed a new equation to predict the shear strength of normal-weight concrete (NWC) and fibre-reinforced polymer (FRP) beams without shear reinforcement. This equation was developed using data from 60 tests on concrete beams reinforced with both carbon fibre-reinforced polymer (CFRP) and glass fibre-reinforced polymer (GFRP) bars. The new equation takes into account the ratio of the elastic modulus of the FRP bars to that of steel reinforcement, the shear span-to-depth ratio, and the flexural reinforcement ratio. Additionally, the applicability of the proposed equation to lightweight concrete (LWC) and FRP beams was investigated using tests on 24 LWC beams in a three-point bending scheme. The concrete shear strengths of the LWC/FRP beams were found to be approximately 75% of the strengths predicted by the proposed equation for the NWC beams. Moreover, when applying a reduction factor of 0.85, the proposed equation also demonstrated promising results for the shear strength of GFRP-reinforced lightweight sand concrete slabs.

The primary aim of the experimental study conducted by Vakili et al. [40] was to examine how various types of fibres—namely glass, polypropylene, and steel—affect the shear strength of lightweight concrete (LWC) beams reinforced with fibre-reinforced polymers (FRPs). Since adding fibres can modify the properties of concrete, we have included the results of this study in our analysis. In the experiment, eight LWC beams were reinforced with two glass fibre-reinforced polymer (GFRP) bars without shear reinforcement and were tested to failure under a four-point bending setup. The results indicated that the addition of fibres to the LWC significantly improved its shear strength and increased the maximum load-carrying capacity by 55% to 233% compared to LWC beams without fibres. Additionally, shear strength correction factors for LWC/FRP beams with and without added fibres were calculated by comparing the experimental results with four specific formulas based on design guidelines and codes: [29,32], and the formulas that have since been replaced by [37,39]. The shear strength correction factor, calculated using the formula α = V_exp_/V_code_, demonstrated the impact of the added fibres in the LWC mix. The factor ranged from 1.31 to 1.82, depending on the type of fibre used.

Bengar et al. [17] investigated the shear behaviour of eight reinforced concrete beams without transverse reinforcement to understand how different concrete types (normal-weight concrete, NWC, versus lightweight concrete, LWC) and reinforcement types (steel versus glass fibre-reinforced polymer, GFRP) affect shear strength. The researchers conducted a four-point bending test. They found that the shear strength of the LWC beams reinforced with GFRP was approximately 50% lower than that of the corresponding NWC beams reinforced with steel bars. However, the shear strength of the LWC/GFRP beams was about 7% higher than that of the LWC beams with steel reinforcement. Additionally, the shear strength results from the tests were compared with predictions made using equations from various codes and studies in the literature. This comparison revealed a good alignment between the experimental results for the LWC/GFRP beams and the predictions provided by the Canadian code [33].

Wiater and Siwowski [19] conducted tests on seven concrete slabs reinforced with GFRP bars under four-point bending. Of these slabs, three were made of normal-weight concrete (NWC), while four were constructed from lightweight concrete (LWC). The results were analysed based on serviceability criteria, including crack width and deflection, as well as ultimate capacity and mode of failure. The NWC slabs failed in flexure, while the LWC slabs typically failed in shear. This was attributed to their lower strength and the use of coarse lightweight aggregate that was twice the size of the aggregate used in the NWC mix. In the LWC slabs, an increase in the reinforcement ratio by 40% resulted in a roughly 10% increase in ultimate shear strength. The test results were then compared to values predicted by the ACI code [38], which has now been replaced by the new ACI code [36]. The research highlighted significant disparities between the test results and the ACI code predictions, with LWC slabs showing greater discrepancies compared to NWC slabs, which had smaller differences. The current ACI shear model significantly underestimates the shear strength of the LWC/FRP slabs, with the predicted maximum shear force being, on average, 67% lower than the experimental values. This discrepancy was a primary motivation for the authors’ pursuit of more accurate calculation methods, and this paper presents partial findings from their ongoing studies.

Mehany et al. [18] investigated the shear performance of lightweight self-consolidating concrete (LWSCC) elements reinforced with basalt fibre-reinforced polymer (BFRP) bars. The anti-corrosion properties of BFRP bars, combined with the benefits of LWSCC, motivated this study to evaluate the behaviour of these elements under shear forces. Five LWSCC beams reinforced with BFRP bars were tested to failure using a four-point bending method. The test results revealed an increase in both the concrete shear strength of the LWSCC/BFRP beams and the axial stiffness of the longitudinal BFRP reinforcing bars. Furthermore, the experimental results were compared with shear strength predictions based on the provisions outlined in various standards. By applying a 0.75 concrete density reduction factor in the CSA code [33] shear equation to account for the influence of concrete density, a more accurate value for the concrete shear strength was achieved. Additionally, using a 0.8 concrete density reduction factor in the ACI [38] design equation provided a suitable level of conservatism when compared to normal-weight concrete (NWC) beams.

**Figure 1:**
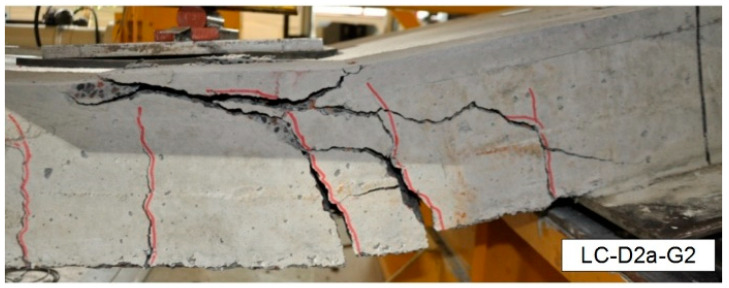
Typical shear failure mode of tested specimens [22].

After reviewing the aforementioned papers, detailed data has been compiled in Table 1 for the 50 LWC/FRP elements tested to experimentally determine their shear strength. Table 1 provides the dimensions of the tested elements, the material characteristics of the lightweight concrete (LWC) and FRP bars, as well as the experimentally determined shear strength. The symbols used in Table 1 are explained at the end of the paper. Additionally, Figure 1 illustrates the typical failure modes observed in the tested elements. The review indicates that the failure mode of the specimens was characterised by shear failure, which began with a critical diagonal shear crack, followed by the crushing of concrete in the compression zone. It corresponds to the shear strength determined based on experimental studies, as in Table 1. The experimentally determined shear strengths, as listed in Table 1, were used to verify the selected code procedures based on the type of structural element, lightweight concrete, and FRP composite bars.

Of the 50 test specimens listed in Table 1, nine are slab specimens and 41 are beam specimens. Unidirectional bending slabs were studied in two cases, and the tested specimens had similar dimensions with a thickness of 0.18–0.24 m and a total length of approximately 2.4 m. The remaining works tested beams with lengths ranging from 1.4 m to 2.60 m, a minimum rectangular cross-section of 0.10 × 0.20 m, and a maximum cross-section of 0.24 × 0.63 m. The maximum size of the beams is relatively small. It is worth mentioning the limitations of the database, as aspects such as the size effect will not be adequately captured, and the variability of results will increase for small member sizes.

The test specimens summarised in Table 1 were made of three different lightweight concretes. The basic material parameters used in the code calculations for these concretes are also summarised in Table 1. Most specimens (in four works) were made of lightweight sand concrete with a density of 1530–1970 kg/m^3^, compressive strength of 21–75 MPa, and a maximum fine aggregate dimension of 8–15 mm (normal-weight sand) and lightweight coarse aggregate. All-lightweight concrete with a density of 1800 kg/m^3^, compressive strength of 18–27 MPa, and a maximum fine aggregate dimension of 15 mm (lightweight sand) and lightweight coarse aggregate was used only in one study [28]. Similarly, only one study utilised fibre-reinforced lightweight concrete with a density of 1520–1680 kg/m^3^, a compressive strength of 32–41 MPa, and a maximum fine aggregate dimension of 9.5 mm [40]. This lightweight sand concrete also contained micro glass fibres, micro polypropylene fibres, and macro steel fibres. Finally, the studies presented in Table 1 utilised three different types of FRP composite bars. Most of the research was conducted on LWC elements reinforced with GFRP bars, which have an elastic modulus of 40–66 GPa. In one study, BFRP bars were used with an elastic modulus of 63–65 GPa [18]. Similarly, one test was partially performed on CFRP bar-reinforced elements with an elastic modulus of 146–148 GPa [28]. A direct comparison of the effects of different types of FRP bars on the shear strength of the elements was carried out only in the work [28] (GFRP and CFRP). However, its authors did not reveal differences in the failure mode of elements, but only differences in the stiffness of beams due to different moduli of elasticity of FRP bars. In the other studies reviewed, reinforcing bars were not differentiated, so it is not possible to assess their effect on failure mode in detail. 

## 3. Review of Provision Documents

The selected design formulas for calculating the shear strength (V_code_) of concrete elements reinforced with FRP composite bars are summarised in Table 2, presented in chronological order. In Table 2, the procedures used in eight provision documents (codes, guidelines, manuals, etc.) are given: Japanese [29], British [30], Italian [31], two Canadian [32,33], two American [34,36], and European [35]. The MCFT (modified compression field theory) method presented in the Canadian bridge standard CSA [41] was omitted due to the need to determine the internal forces M and V in the cross-section of the element. In the remaining formulas, the assumption of the relation between the internal forces M = V·a was made. The notation used in the formulas in Table 2 is listed at the end of the paper.

The parameters considered in the individual calculations are summarised in Table 3. As can be seen from this table, most of the various parameters are included in the formula according to CEN [35]. In all analysed formulas, the following parameters were taken into account: the width of the element (b), the height of the section (d), and the compressive strength of the concrete (f_c_). In all formulas except ISIS [35], the FRP composite longitudinal reinforcement ratio (*ρ*_f_) is also considered. The FRP composite’s elastic modulus (E_f_) is not included in AASHTO [34] only, as this code applies only to GFRP reinforcement. Reinforcing steel elastic modulus (E_s_) is not used directly in the AASHTO [34] formulas. Shear slenderness (a/d), also expressed as (M/V), is included in AASHTO [34] and CEN [35]. The total height of section (h) was taken into account only in CSA [33], the concrete elastic modulus (E_c_) only in the ACI code [36], and the maximum aggregate dimension (a_g_) only in the European CEN [35]. All standards and guidelines except AASHTO [34] give design procedures generally for FRP bars, without a detailed distinction between the types of bars. The definition of an FRP bar typically lists carbon, aramid, and glass fibres (without basalt), and design procedures only consider Young’s modulus of the bars. The AASHTO standard, as indicated by its title, applies only to GFRP bars and indirectly includes their modulus in the formulas.

The shear strength calculation (V_code_) results according to various code formulas listed in Table 2 are presented in Table 4. The calculation results do not account for material safety factors (γ_m_, whose values in the relevant formulas were assumed to be 1.00). However, they consider the reduction factors for lightweight concrete. The reduction factors exist only in two Canadian codes [32,33] (0.85 for sand concrete and 0.75 for the remaining lightweight concretes). On the other hand, the new ACI code [36] does not apply to elements made of lightweight concrete due to the lack of tests and data in this area. In some codes, the value of the elastic modulus of concrete, E_c_, is necessary to determine the shear strength Vcode. If the value of E_c_ was not determined in the tests (see Table 3), the calculation assumed the modulus of elasticity calculated according to CEN [35] as for ordinary concrete ($E_{c}=\;22\cdot {\left(0.1\cdot f_{c}\right)}^{0.3}$). The reduction in the E_c_ modulus due to the lower density of lightweight concrete is not considered. Furthermore, in the studies by Vakilli et al. [40] and the authors [14], the compressive strength of concrete was determined on cubic samples. In these cases, to determine the cylindrical compressive strength adopted in the calculations, the conversion factor 0.91·f_c.cube_ was used according to CEN [35].

## 4. Code vs. Experiment–Comparison and Discussion

**Figure 2:**
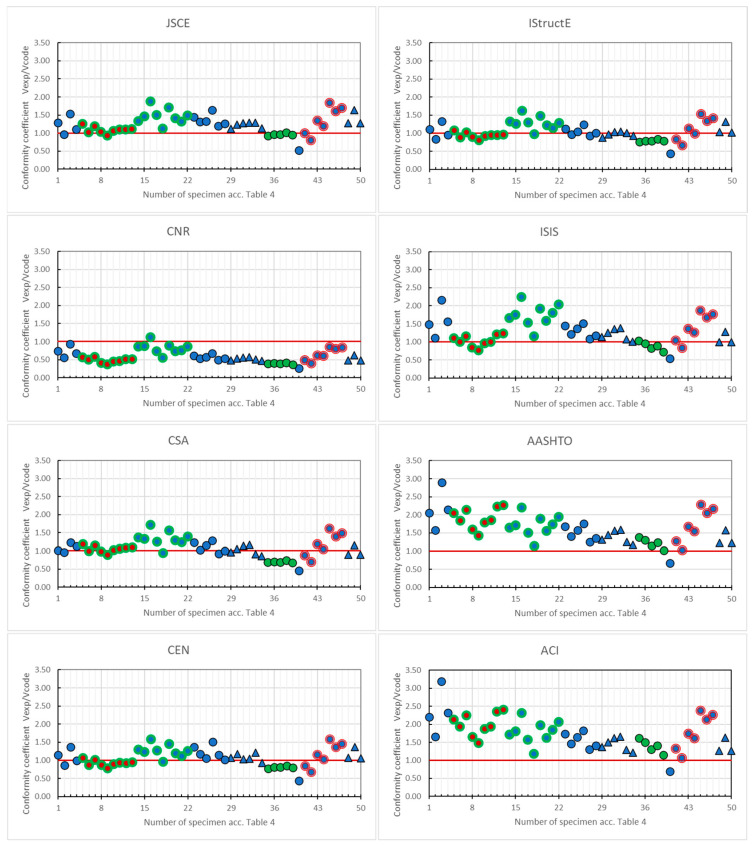
The conformity coefficient (V_exp_/V_code_) for the various code procedures [29,30,31,32,33,34,35,36].

The results of comparing shear strength calculations (V_code_) according to various code procedures with values obtained from experimental research (V_exp_) are presented qualitatively and quantitatively in Figure 2 and Table 5, respectively. The compliance of the code procedures with the test results was assessed based on the conformity coefficient, which is defined as the mean ratio between the experimental and calculated values (V_exp_/V_code_). The value of the conformity coefficient at the level of 0.90 ÷ 1.10 was assumed to be very good (i.e., ±10%). To better understand the influence of individual material variables on the shear strength of the elements, the parameters were distinguished on the plots in Figure 1.

The individual designations are as follows: the type of composite reinforcement (blue-GFRP; red–CFRP; green–BFRP) and the type of lightweight concrete (black marker stroke—sand lightweight concrete; green bold marker stroke–all-lightweight concrete; red double line marker stroke–fibre-reinforced lightweight concrete). The results of the tests of slabs and beams were compiled together because the code procedures for these elements are identical (however, the diagrams distinguish the type of tested elements as well).

Visually evaluating the plots in Figure 2, it can be noted that the calculations performed according to the procedures of the codes IStructE [30] and CEN [35] are the most consistent with the test results. In both codes, the best compliance of the calculation procedures with the test results was obtained for beams made of all-lightweight concrete reinforced with CFRP bars and for lightweight sand concrete slabs reinforced with GFRP bars.

Only a slightly underestimated strength was obtained for the lightweight sand concrete beams reinforced with BFRP bars. On the other hand, the most overestimated strength was obtained in both codes for beams reinforced with GFRP bars, regardless of the type of concrete. In both cases, the degree of underestimation or overestimation of the calculation results varies within ±50%.

The most underestimated values of shear strength are given by the calculation procedures included in both American codes, i.e., the AASHTO [34] and ACI [36]. The most significant non-compliance for all tested elements, regardless of the type of lightweight concrete used or the type of composite reinforcement, is identified through calculations according to the AASHTO code [34]. For most tested elements, the actual shear strength obtained in the tests is more than two times higher than the code shear strength. Interestingly, the most significant discrepancy (up to a four-fold underestimation of strength) was observed for all-lightweight concrete beams reinforced with GFRP bars. However, this underestimation of the shear strength, understandable for safety reasons with relatively new structural elements, seems excessive, which can result in significantly overestimated utilisation of both materials, increased costs, and, as a result, reluctance to use such structural elements. The need to modify the American code in this area (e.g., using the European procedures included in the CEN [35] code) seems obvious.

The code that overestimates the shear strength of almost all structural elements under investigation is the Italian code CNR [32]. For most tested elements, the degree of overestimation of the shear strength according to this code is approximately 50%. On the other hand, the code unifies the shear strength values to the greatest extent, regardless of the type of elements tested and the materials used. From this perspective, the design procedures in this code are the most universal for calculating the shear strength of lightweight concrete structural elements reinforced with composite bars. However, it is necessary to use a reduction factor (also unified), which would ensure greater consistency of the results of calculations according to the CNR code [32] and experimental tests. From the appropriate plot in Figure 2, such a coefficient should be about 0.75.

In addition to the qualitative assessment presented above of individual code procedures based on the results of experimental tests, Table 5 presents the values of the conformity coefficient, defined as the mean ratio between the experimental and calculated values of shear strength (V_exp_/V_code_), enabling a quantitative evaluation of individual procedures. Below are some of the most important conclusions derived from the data in Table 4, considering the division into types of elements, reinforcement, and lightweight concrete.

It is helpful to start by providing the conformity coefficients (V_exp_/V_code_) for the “best practices” indicated in the qualitative assessment as a reference. Analysing the entire population of test results, the best agreement was obtained for the IStructE [30], CSA [33], and CEN [35] procedures, for which the conformity coefficient ranges from 1.04 to 1.08, with a standard deviation ranging from 0.23 to 0.26. Taking into account the type of tested elements, the best agreement for beams was obtained using the same code procedures, with a conformity coefficient in the range of 1.05 to 1.09 and a standard deviation in the range of 0.25 to 0.28. A minor standard deviation was obtained for the CNR procedure [32], i.e., 0.19, but with a conformity coefficient of 0.61. On the other hand, the best conformity coefficient for the slabs was obtained for the IStructE [30] and CSA [33] codes, in the range of 1.00 to 1.10, with a standard deviation of 0.13 to 0.14. It is also worth noting that in the case of the slabs, all calculation procedures yielded a standard deviation of the conformity coefficient ranging from 0.05 to 0.18. However, the significance of this observation is uncertain due to the small number of tested slabs.

Regarding the type of composite reinforcement, the group of tested elements reinforced with GFRP bars had the most significant number. For these elements, the best compatibility of calculation procedures was achieved with the IStructE code [30] at a level of 1.10, with a standard deviation of 0.24. In the case of elements reinforced with CFRP bars, excellent compliance was achieved for the oldest JSCE [29], IStructE [30], ISIS [31], and CSA [33] procedures, with a range of 0.92 to 1.09 and a standard deviation of 0.08 to 0.16. For all calculation procedures involving CFRP reinforcement, the standard deviation of the conformity factor ranged from 0.07 to 0.31; the smallest value was obtained for CNR [32] and the largest for AASHTO [34]. On the other hand, for elements reinforced with BFRP bars, an excellent agreement was obtained for the JSCE [29] procedure in the range of 0.91 ÷ 0.97 with a standard deviation of 0.03 ÷ 0.06.

The last stage of the quantitative analysis involves categorising the type of lightweight concrete, where sand lightweight concrete, all lightweight concrete, and fibre-reinforced concrete are distinguished. For lightweight sand concrete, excellent compliance was obtained for the IStructE [30], CSA [33], and CEN [35] procedures, with conformity coefficients in the range of 0.96 to 1.04 and a standard deviation in the range of 0.19 to 0.23. For all lightweight concretes, the best conformance was obtained using the CEN [35] procedure, with conformity coefficients of 0.109 and a standard deviation of 0.22. For fibre-reinforced lightweight concrete, excellent compliance was obtained for the IStructE [30] and CEN [35] procedures: the compliance at the level of 1.13 ÷ 1.16 with a standard deviation in the range of 0.32 ÷ 0.33. However, it should be noted that results for all-lightweight and fibre-reinforced lightweight concretes were obtained only from a few tests in two single research studies.

Taking into account the results presented above of the qualitative and quantitative comparative analysis of the various standard procedures for calculating the shear strength of lightweight concrete structural elements reinforced with composite bars, there is great difficulty in indicating the procedures most consistent with the experimental results and thus unifying the standard calculation procedures for all LWC/FRP structural elements. This is mainly due to the numerous research variables (two types of structural elements, three types of reinforcement, and three types of concretes), each of which requires modifications to empirical calculation formulas in a specific manner. The analysis shows that the three procedures that most often show good compliance and the lowest dispersion are the IStructE [30], CSA [33], and CEN [35] codes. For these three codes, the mean compliance coefficients and the relevant standard deviations are 1.04/0.23, 1.08/0.26, and 1.08/0.24. A slight overestimate of shear strength in these three codes (4–8%) is beneficial for safety reasons. However, it does not lead to oversized structural elements and does not require the use of relatively expensive materials. Special emphasis should be placed on the presence of a new European standard in this small group (CEN [35]), which is the most recent one, meaning that the calculation procedure was developed based on the latest research results in the field. Importantly, it is consistent with the entire system of the new edition of European construction standards, known as the Eurocodes. The procedures included in the American codes (AASHTO [34], ACI [36]), for which the following results were obtained: 1.63/0.43 and 1.72/0.45, have been much more overestimated.

To summarise the above discussion, the best procedure today for calculating the shear strength of LWC/FRP structural elements is the procedure outlined in the European Standard CEN [35]. With the publication of the results of subsequent tests on the LWC/FRP elements (beams, slabs) under flexure, the European procedure can be further modified and improved to achieve better conformance, especially smaller spreads. The most justified is to modify the influence of individual input parameters (f_c_, *ρ*_f_, E_f_) in the V_code_ formula (Table 2, item 7). The impact of these parameters on the Vcode value can be evaluated according to the proposal by Gao and Zhang [42]. Another direction for modifying and improving the V_code_ formula may be the use of artificial intelligence, such as an artificial neural network (ANN). However, the existing database of single test results of the LWC/FRP elements (50 items) is still too small, so it is worth considering using the database for the NWC/FRP created by Cholostiakow et al. [43], containing 326 items from the years 1993–2016, in which it would be possible to adjust, e.g., the density of concrete. Regardless of the choice of the procedure for its modification based on the entire experimental database, it does not seem justified, as proposed in some codes, to modify and differentiate design procedures depending on material parameters (LWC type, FRP type), as it would overcomplicate the design process of the LWC/FRP elements.

The economics in the design of LWC/FRP reinforced elements are indirectly demonstrated by the compliance of the experimental results with the design calculations (Figure 1). The greater the non-compliance, the greater the overestimation or underestimation of the shear resistance of the element. In the case of overestimation, we are dealing with an excessive suspension of project costs. In this way, we can determine which of the standards is the “most economical”. The relevant conclusion was included in the paper’s conclusion. However, it should be noted that underestimation can reduce the safety of the structure.

## 5. Conclusions

This paper investigates the optimal code procedure (or formula) for predicting the shear strength of lightweight concrete structural elements, specifically beams and slabs, that are reinforced with FRP bars and do not use stirrups. An experimental database of 50 FRP bar-reinforced lightweight concrete elements without stirrups has been established to assess the effectiveness of the code formulas by comparing the calculated shear strengths against the experimental results. To the best of the authors’ knowledge, no studies on the shear behaviour of lightweight concrete elements reinforced with FRP have been published since 2023. The main conclusions of this research can be summarised as follows:

- Identifying the calculation procedures that best align with experimental results for lightweight concrete (LWC) and fibre-reinforced polymer (FRP) structural elements presents significant challenges. This complexity arises from the numerous research variables, each of which necessitates specific modifications to empirical calculation formulas.
- The analysis indicates that three procedures consistently demonstrate good compliance and the lowest variability: those outlined in the IStructE [30], CSA [33], and CEN [35] codes. In contrast, the procedures found in American codes, such as AASHTO [34] and ACI [36], tend to overestimate results significantly.
- Moreover, the findings suggest that adopting a single code procedure for the entire population of test results is more effective than modifying separate procedures for different lightweight concretes and FRP bars to enhance compliance.
- Among the analysed procedures, the most recent CEN [35] method aligns best with the experimental results related to the shear strength of FRP bar-reinforced lightweight concrete elements that lack stirrups. The conformity coefficient (V_exp_/V_code_) for the CEN [35] procedure is very close to 1.0. Consequently, the authors did not propose a new formula, despite noting that none of the existing formulas is entirely adequate.

Considering the limitations of the current database, the authors suggest the following avenues for further research:

- One potential improvement to the CEN procedure is to assess how certain parameters (f_c_, *ρ*_f_, E_f_) affect the V_code_ value. This evaluation could help achieve higher conformity coefficient values.
- Additionally, artificial neural networks (ANNs) could be utilised to enhance the procedure. However, the existing database of single test results for LWC/FRP elements, which currently contains only 50 entries, needs to be expanded.
- Moreover, it may be beneficial to use the database for NWC/FRP elements, which includes 326 entries collected from 1993 to 2016. This larger dataset could allow for adjustments, such as modifying the density of concrete.

## Figures and Tables

**Table 1:** Data from 50 LWC/FRP elements tested to experimentally determine the shear strength.

Source, Year	Specimen	Dimensions					LWC			FRP				Shear Strength
		l_t_ [m]	b [mm]	h [mm]	a [mm]	d [mm]	ρ_lc_ [kg/m^3^]	f_c_ [MPa]	Fine Aggregate	Reinf. Type	E_f_ [GPa]	d_max_ [mm]	ρ_f_ [%]	V_exp_ [kN]
Bengar et al. [17], 2021	G-L-D12-2.5	2.00	150	250	500	200	1824	21.0	sand	GFRP	66.40	10.00	0.750	20.00
	G-L-D12-4	2.00	150	250	800	200	1824	21.0	sand	GFRP	66.40	10.00	0.750	15.00
	G-L-D16-2.5	2.00	150	250	500	200	1824	21.0	sand	GFRP	66.40	10.00	1.340	29.00
	G-L-D16-4	2.00	150	250	800	200	1824	21.0	sand	GFRP	66.40	10.00	1.340	21.00
Kim & Jang [28], 2014	C-L-18-R1-1	2.20	200	250	646.5	215.5	1800	18.0	lightwieght	CFRP	146.20	15.00	0.331	25.80
	C-L-18-R2-1	2.20	150	250	646.5	215.5	1800	18.0	lightwieght	CFRP	146.20	15.00	0.441	17.50
	C-L-18-R2-2	2.20	150	250	646.5	215.5	1800	18.0	lightwieght	CFRP	146.20	15.00	0.441	20.30
	C-L-27-R1-1	2.20	200	250	646.5	215.5	1800	27.0	lightwieght	CFRP	146.20	15.00	0.331	24.40
	C-L-27-R1-2	2.20	200	250	646.5	215.5	1800	27.0	lightwieght	CFRP	146.20	15.00	0.331	21.90
	C-L-27-R2-1	2.20	150	250	646.5	215.5	1800	27.0	lightwieght	CFRP	146.20	15.00	0.441	20.70
	C-L-27-R2-2	2.20	150	250	646.5	215.5	1800	27.0	lightwieght	CFRP	146.20	15.00	0.441	21.50
	C-L-27-R3-1	2.20	150	250	640.5	213.5	1800	27.0	lightwieght	CFRP	147.90	15.00	0.791	25.90
	C-L-27-R3-2	2.20	150	250	640.5	213.5	1800	27.0	lightwieght	CFRP	147.90	15.00	0.791	26.40
	G-L-18-R1-1	2.20	200	250	646.5	215.5	1800	18.0	lightwieght	GFRP	41.00	15.00	0.331	20.70
	G-L-18-R2-1	2.20	150	250	646.5	215.5	1800	18.0	lightwieght	GFRP	41.00	15.00	0.441	16.30
	G-L-18-R2-2	2.20	150	250	646.5	215.5	1800	18.0	lightwieght	GFRP	41.00	15.00	0.441	20.90
	G-L-27-R1-1	2.20	200	250	646.5	215.5	1800	27.0	lightwieght	GFRP	41.00	15.00	0.331	23.20
	G-L-27-R1-2	2.20	200	250	646.5	215.5	1800	27.0	lightwieght	GFRP	41.00	15.00	0.331	17.50
	G-L-27-R2-1	2.20	150	250	646.5	215.5	1800	27.0	lightwieght	GFRP	41.00	15.00	0.441	21.90
	G-L-27-R2-2	2.20	150	250	646.5	215.5	1800	27.0	lightwieght	GFRP	41.00	15.00	0.441	18.00
	G-L-27-R3-1	2.20	150	250	640.5	213.5	1800	27.0	lightwieght	GFRP	40.00	15.00	0.791	20.20
	G-L-27-R3-2	2.20	150	250	640.5	213.5	1800	27.0	lightwieght	GFRP	40.00	15.00	0.791	22.70
Liu & Pantelides [16], 2013	#4 B1LW	2.44	610	235	1220	202	1970	63.0	sand	GFRP	43.40	12.70	0.940	112.37
	#5 B1LW	2.44	610	235	1220	202	1970	75.0	sand	GFRP	43.40	12.70	0.940	102.72
	#6 B2LW	2.44	610	235	1220	202	1970	60.0	sand	GFRP	43.40	12.70	0.940	103.12
	#7 B1LW	2.44	610	235	1220	202	1970	68.0	sand	GFRP	43.40	12.70	0.830	122.15
	#10 B1LW	2.90	610	273	1450	240	1970	63.0	sand	GFRP	43.40	12.70	0.790	99.80
	#11 B2LW	2.90	610	273	1450	240	1970	60.0	sand	GFRP	43.40	12.70	0.790	105.36
	#14 B1LW	2.44	1830	235	1220	202	1970	63.0	sand	GFRP	43.40	12.70	0.960	264.74
	#15 B1LW	2.44	1830	235	1220	202	1970	63.0	sand	GFRP	43.40	12.70	0.960	290.16
	#16 B2LW	2.44	1830	235	1220	202	1970	57.0	sand	GFRP	43.40	12.70	0.960	298.99
	#17 B2LW	2.44	1830	235	1220	202	1970	56.0	sand	GFRP	43.40	12.70	0.960	302.52
	#19 B1LWD	2.44	1830	235	1220	202	1970	63.0	sand	GFRP	43.40	12.70	0.540	249.23
	#20 B2LWD	2.44	1830	235	1220	202	1970	56.0	sand	GFRP	43.40	12.70	0.540	221.14
Mehany et al. [18], 2022	LSBI-1.26	2.60	200	400	1000	333.3	1800	54.0	sand	BFRP	63.70	15.00	1.260	44.85
	LSBI-0.83	2.60	200	400	1000	333.3	1800	54.0	sand	BFRP	63.70	15.00	0.830	39.05
	LSBII-0.86	2.60	200	400	1000	333.3	1800	54.0	sand	BFRP	64.80	15.00	0.860	42.10
	LSBII-0.58	2.60	200	400	1000	333.3	1800	54.0	sand	BFRP	64.80	15.00	0.580	34.45
Vakili et al. [40], 2019	LWC	1.40	100	200	550	170	1530	31.9	sand	GFRP	49.40	9.50	0.590	4.41
	GLWC	1.40	100	200	550	170	1552	33.2	sand + fibre	GFRP	49.40	9.50	0.590	8.58
	PLWC	1.40	100	200	550	170	1521	32.8	sand + fibre	GFRP	49.40	9.50	0.590	6.86
	SLWC	1.40	100	200	550	170	1673	41.0	sand + fibre	GFRP	49.40	9.50	0.590	12.50
	GPLWC	1.40	100	200	550	170	1565	30.9	sand + fibre	GFRP	49.40	9.50	0.590	10.05
	GSLWC	1.40	100	200	550	170	1680	40.0	sand + fibre	GFRP	49.40	9.50	0.590	16.92
	PSLWC	1.40	100	200	550	170	1588	32.8	sand + fibre	GFRP	49.40	9.50	0.590	13.73
	GPSLWC	1.40	100	200	550	170	1620	33.7	sand + fibre	GFRP	49.40	9.50	0.590	14.71
Wiater & Siwowski [19], 2020	LC-D2a-G1	2.40	1000	180	600	151	1857	42.7	sand	GFRP	49.00	8.00	0.280	82.40
	LC-D2a-G2	2.40	1000	180	600	151	1857	42.7	sand	GFRP	49.00	8.00	0.280	106.20
	LC-D2a-G0	2.40	1000	180	600	151	1857	42.7	sand	GFRP	49.00	8.00	0.280	82.20

**Table 2:** The selected design formulas for calculating the shear strength (V_code_) of the concrete elements reinforced with FRP composite bars.

No.	Code	Formulas for Calculating V_code_
1.	JSCE [29]	$V_{c}=\beta_{d}\cdot \beta_{p}\cdot \beta_{n}\cdot f_{vcd}\cdot b\cdot d$ $\beta_{d}=\sqrt[{4}]{\frac{1}{d\left[m\right]}}\;\leq 1.5$ $\beta_{p}=\sqrt[{3}]{100\cdot \rho_{f}\cdot \frac{E_{f}}{E_{s}}}\leq 1.5$ $\beta_{n}=1.0$ no axial load $f_{vcd}=0.2\cdot \sqrt[{3}]{f_{c}}\leq 0.72\frac{N}{mm^{2}}$
2.	IStructE [30]	$V_{c}=0.79\cdot {\left(100\cdot \rho_{f}\cdot \frac{E_{f}}{E_{s}}\right)}^{\frac{1}{3}}\cdot {\left(\frac{400}{d}\right)}^{\frac{1}{4}}\cdot {\left(\frac{f_{cu}}{25}\right)}^{\frac{1}{3}}\cdot b\cdot d$ $f_{cu}=1.25\cdot f_{c}$
3.	CNR [31]	$V_{c}=min(V_{ct};V_{max})$ $V_{ct}=1.3\cdot {\left(\frac{E_{f}}{E_{s}}\right)}^{\frac{1}{2}}\cdot \tau_{Rd}\cdot k_{d}\cdot (1.2+40\cdot \rho_{f})\cdot b\cdot d$ $V_{max}=0.5\cdot v_{1}\cdot f_{c}\cdot b\cdot 0.9\cdot d$ $\tau_{Rd}=0.25\cdot f_{ct}$ $k_{d}=1.6-d[m]\geq 1.0$ $v_{1}=\left\{\begin{array}{ccc} 0.6 & for & f_{c}\leq 60\;[MPa]\\ 0.9-\frac{f_{c}}{200}\geq 0.5 & for & f_{c}>60\;[MPa] \end{array}\right.$ $1.3\cdot {\left(\frac{E_{f}}{E_{s}}\right)}^{\frac{1}{2}}\leq 1.0$
4.	ISIS [32]	For *d* < 300 mm: $V_{c}=0.2\cdot \sqrt{f_{c}}\cdot b\cdot d\cdot \sqrt{\frac{E_{f}}{E_{s}}}$ For *d* > 300 mm: $V_{c}=\left(\frac{260}{1000-d}\right)\cdot \sqrt{f_{c}}\cdot b\cdot d\cdot \sqrt{\frac{E_{f}}{E_{s}}}\geq 0.1\cdot \sqrt{f_{c}}\cdot b\cdot d\cdot \sqrt{\frac{E_{f}}{E_{s}}}$
5.	CSA [33]	$V_{c}=0.05\cdot k_{m}\cdot k_{r}\cdot \sqrt[{3}]{f_{c}}\cdot b\cdot d_{v}$ $k_{m}=\sqrt{\frac{V\cdot d}{M}}\leq 1.0$ $k_{r}=1+{\left(E_{f}\cdot \rho_{f}\right)}^{\frac{1}{3}}$ $d_{v}=max(0.9\cdot d;0.72\cdot h)$ $0.11\cdot \sqrt{f_{c}}\cdot b\cdot d_{v}\leq V_{c}\leq 0.22\cdot \sqrt{f_{c}}\cdot b\cdot d_{v}$ $f_{c}<60\;[MPa]$
6.	AASHTO [34]	$V_{c}=\left(0.0676\cdot \;\sqrt{f_{c}}+4.6\;\cdot \rho_{f}\cdot \frac{d}{a}\right)\cdot b\cdot d\leq 0.126\cdot \sqrt{f_{c}}\;\cdot b\cdot d$
7.	CEN [35]	$V_{c}={\left(100\cdot \rho_{f}\cdot \frac{E_{f}}{E_{s}}\cdot f_{c}\cdot \frac{d_{dg}}{a_{v}}\right)}^{\frac{1}{3}}\cdot b\cdot d$ $d_{dg}=\left\{\begin{array}{ccc} min(16+a_{g}\cdot {\left(\frac{60}{f_{c}}\right)}^{2};40) & for & f_{c}>\;60\;MPa\\ min(16+a_{g};40) & for & f_{c}\leq \;60MPa \end{array}\right.$ $a_{v}=\max\left(\frac{M}{V}\;;2.5\cdot d\right)$ $V_{c}\geq V_{c.min}$ $V_{c.min}=6\cdot \sqrt{\frac{f_{c}}{500}\cdot \frac{E_{f}}{E_{s}}\cdot \frac{d_{dg}}{d}}\cdot b\cdot d$
8.	ACI [36]	$V_{c}=\max\left(\;0.42\cdot \lambda_{s}\cdot k_{cr}\cdot \sqrt{f_{c}}\cdot b\cdot d;\;0.066\cdot \lambda_{s}\cdot \sqrt{f_{c}}\cdot b\cdot d\right)$ $k_{cr}=\sqrt{2\cdot \rho_{f}\cdot \frac{E_{f}}{E_{c}}+{\left(\rho_{f}\cdot \frac{E_{f}}{E_{c}}\right)}^{2}}-\rho_{f}\cdot \frac{E_{f}}{E_{c}}$ $\lambda_{s}=\sqrt{\frac{2}{1+0.004\cdot d}}\leq 1.0$

**Table 3:** Parameters taken into account in various code formulas.

Code/Parameters	b	d	h	$\left(\frac{a}{d}\right)$ $\;\mathrm{or}\;\left(\frac{M}{V}\right)$	f_c_	E_c_	E_f_	E_s_	*ρ*_f_	a_g_
JSCE [29]	+	+	-	-	+	-	+	+	+	-
IStructE [30]	+	+	-	-	+	-	+	+	+	-
CNR [31]	+	+	-	-	+	-	+	+	+	-
ISIS [32]	+	+	-	-	+	-	+	+	-	-
CSA [33]	+	+	+	+	+	-	+	-	+	-
AASHTO [34]	+	+	-	+	+	-	-	-	+	-
CEN [35]	+	+	-	+	+	-	+	+	+	+
ACI [36]	+	+	-	-	+	+	+	-	+	-

“+” parameter is taken into account in code formula; “-” parameter is not take into account in code formula.

**Table 4:** Results of the shear strength calculations (V_code_) according to various code formulas.

Source	No.	Specimen	V_exp_	Shear Strength V_code_ [kN] According to Code Formulas ^1^							
			[kN]	JSCE [29]	Istructe [30]	ISIS [31]	CNR [32]	CSA [33]	AASHTO [34]	CEN [35]	ACI [36]
Bengar et al. [17]	1	G-L-D12-2.5	20.00	15.57	18.02	13.46	26.94	19.86	9.71	19.44	9.07
	2	G-L-D12-4	15.00	15.57	18.02	13.46	26.94	15.70	9.55	16.62	9.07
	3	G-L-D16-2.5	29.00	18.90	21.87	13.46	31.18	23.62	10.03	23.58	9.07
	4	G-L-D16-4	21.00	18.90	21.87	13.46	31.18	18.67	9.76	20.16	9.07
Kim & Jang [28]	5	C-L-18-R1-1	25.80	20.66	23.91	23.45	45.52	21.65	12.58	25.57	12.07
	6	C-L-18-R2-1	17.50	17.05	19.73	17.59	35.27	17.68	9.49	21.10	9.05
	7	C-L-18-R2-2	20.30	17.05	19.73	17.59	35.27	17.68	9.49	21.10	9.05
	8	C-L-27-R1-1	24.40	23.65	27.37	28.73	59.65	24.78	15.36	29.27	14.78
	9	C-L-27-R1-2	21.90	23.65	27.37	28.73	59.65	24.78	15.36	29.27	14.78
	10	C-L-27-R2-1	20.70	19.52	22.59	21.54	46.21	20.24	11.57	24.16	11.09
	11	C-L-27-R2-2	21.50	19.52	22.59	21.54	46.21	20.24	11.57	24.16	11.09
	12	C-L-27-R3-1	25.90	23.64	27.36	21.47	50.81	24.00	11.64	29.28	10.98
	13	C-L-27-R3-2	26.40	23.64	27.36	21.47	50.81	24.00	11.64	29.28	10.98
	14	G-L-18-R1-1	20.70	15.52	15.56	12.42	24.11	15.08	12.58	16.74	12.07
	15	G-L-18-R2-1	16.30	11.16	12.92	9.32	18.68	12.20	9.49	13.81	9.05
	16	G-L-18-R2-2	20.90	11.16	12.92	9.32	18.68	12.20	9.49	13.81	9.05
	17	G-L-27-R1-1	23.20	15.48	17.92	15.21	31.59	18.47	15.36	19.16	14.78
	18	G-L-27-R1-2	17.50	15.48	17.92	15.21	31.59	18.47	15.36	19.16	14.78
	19	G-L-27-R2-1	21.90	12.78	14.79	11.41	24.47	13.97	11.57	15.81	11.09
	20	G-L-27-R2-2	18.00	12.78	14.79	11.41	24.47	13.97	11.57	15.81	11.09
	21	G-L-27-R3-1	20.20	15.29	17.69	11.16	26.42	16.25	11.64	18.94	10.98
	22	G-L-27-R3-2	22.70	15.29	17.69	11.16	26.42	16.25	11.64	18.94	10.98
Liu & Pantelides [16]	23	#4 B1LW	112.37	77.90	99.64	77.45	182.28	91.44	67.00	68.33	64.55
	24	#5 B1LW	102.72	77.90	105.61	84.51	194.31	99.77	73.02	73.34	70.43
	25	#6 B2LW	103.12	77.90	98.04	75.58	178.99	89.24	65.40	81.36	62.99
	26	#7 B1LW	122.15	74.73	98.06	80.47	182.26	95.00	69.47	67.21	67.06
	27	#10 B1LW	99.80	83.66	107.01	92.02	202.66	108.65	79.43	72.33	76.69
	28	#11 B2LW	105.36	83.66	105.28	89.80	199.00	106.03	77.54	86.12	74.84
	29	#14 B1LW	264.74	235.35	301.04	232.36	549.60	274.34	201.05	206.45	193.65
	30	#15 B1LW	290.16	235.35	301.04	232.36	549.60	274.34	201.05	206.45	193.65
	31	#16 B2LW	298.99	235.53	291.16	221.02	529.38	260.95	191.37	241.65	184.20
	32	#17 B2LW	302.52	235.35	289.45	219.06	525.85	258.65	189.70	240.22	182.57
	33	#19 B1LWD	249.23	194.27	248.50	232.36	491.31	274.34	199.86	170.42	193.65
	34	#20 B2LWD	221.14	194.27	238.93	219.06	470.08	258.65	188.52	198.30	182.57
Mehany et al. [18]	35	LSBI-1.75	48.40	51.99	63.17	47.01	123.16	70.30	34.91	65.14	29.93
	36	LSBI-1.26	44.85	46.60	56.62	47.01	110.45	63.64	34.40	58.39	29.93
	37	LSBI-0.83	39.05	40.54	49.26	47.01	99.30	56.18	33.97	50.80	29.93
	38	LSBII-0.86	42.10	41.26	50.13	47.40	100.94	57.06	34.00	51.70	29.93
	39	LSBII-0.58	34.45	36.18	43.96	47.40	93.62	50.80	33.71	45.34	29.93
Vakili et al. [40]	40	LWC	4.41	8.51	10.22	8.11	17.00	9.72	6.63	10.19	6.34
	41	GLWC	8.58	8.63	10.37	8.28	17.48	9.85	6.77	10.33	6.47
	42	PLWC	6.86	8.59	10.32	8.22	17.32	9.81	6.72	10.28	6.43
	43	SLWC	12.50	9.25	11.11	9.19	20.10	10.57	7.50	11.08	7.18
	44	GPLWC	10.05	8.43	10.12	7.99	16.67	9.62	6.53	10.09	6.24
	45	GSLWC	16.92	9.18	11.03	9.09	19.80	10.49	7.41	10.99	7.10
	46	PSLWC	13.73	8.59	10.32	8.22	17.32	9.81	6.72	10.28	6.43
	47	GPSLWC	14.71	8.67	10.41	8.34	17.64	9.89	6.81	10.38	6.51
Wiater & Siwowski [19]	48	LC-D2a-G1	82.40	64.82	80.22	83.03	169.25	92.26	67.19	73.89	65.12
	49	LC-D2a-G2	106.20	64.82	80.22	83.03	169.25	92.26	67.19	73.89	65.12
	50	LC-D2a-G0	82.20	64.82	80.22	83.03	169.25	92.26	67.19	73.89	65.12

^1^ The calculation results do not take into account material safety factors γ_m_ but consider the reduction factors for lightweight concrete.

**Table 5:** The conformity coefficient (V_exp_/V_code_) for various code procedures.

Code			JSCE [29]	IStructE [30]	ISIS [31]	CNR [32]	CSA [33]	AASHTO [34]	CEN [35]	ACI [36]
Entire population		MAX	1.87	1.62	2.24	1.12	1.71	2.89	1.59	3.20
		MIN	0.52	0.43	0.54	0.26	0.45	0.67	0.44	0.70
		AVG	1.25	**1.04**	1.29	0.59	**1.08**	1.63	**1.08**	1.72
		SD	0.27	**0.23**	0.38	0.18	**0.26**	0.42	**0.24**	0.45
Type of tested members	beams	MAX	1.87	1.62	2.24	1.12	1.71	2.89	1.59	3.20
		MIN	0.52	0.43	0.54	0.26	0.45	0.67	0.44	0.70
		AVG	1.24	**1.05**	1.31	0.61	**1.09**	1.68	**1.07**	1.78
		SD	0.29	**0.25**	0.41	0.19	**0.28**	0.44	**0.26**	0.46
	slabs	MAX	1.64	1.32	1.38	0.63	1.17	1.59	1.37	1.66
		MIN	1.12	0.88	0.99	0.47	0.85	1.17	0.93	1.21
		AVG	1.28	**1.02**	1.16	0.53	**1.00**	1.37	1.11	1.42
		SD	0.15	**0.13**	0.16	0.05	**0.13**	0.17	0.13	0.18
Type of reinforcement	GFRP	MAX	1.87	1.62	2.24	1.12	1.71	2.89	1.59	3.20
		MIN	0.52	0.43	0.54	0.26	0.45	0.67	0.44	0.70
		AVG	1.33	**1.10**	1.41	0.65	1.13	1.61	1.15	1.69
		SD	0.28	**0.24**	0.38	0.18	0.26	0.42	0.24	0.47
	CFRP	MAX	1.25	1.08	1.23	0.58	1.19	2.27	1.06	2.40
		MIN	0.93	0.80	0.76	0.37	0.88	1.43	0.79	1.48
		AVG	**1.09**	**0.94**	**1.03**	0.48	**1.05**	1.91	**0.92**	2.00
		SD	**0.09**	**0.08**	**0.16**	0.07	**0.09**	0.29	**0.08**	0.31
	BFRP	MAX	1.02	0.84	1.03	0.42	0.74	1.39	0.86	1.62
		MIN	0.93	0.77	0.73	0.37	0.68	1.02	0.78	1.15
		AVG	**0.97**	0.79	0.89	0.40	0.70	1.22	0.81	1.40
		SD	**0.03**	0.03	0.12	0.02	0.02	0.14	0.03	0.18
Type of lightweight concrete	sand	MAX	1.64	1.33	2.15	0.93	1.29	2.89	1.51	3.20
		MIN	0.52	0.43	0.54	0.26	0.45	0.67	0.44	0.70
		AVG	1.20	**0.97**	1.18	0.53	**0.96**	1.47	**1.04**	1.57
		SD	0.24	**0.19**	0.32	0.14	**0.21**	0.43	**0.23**	0.47
	all lightweight	MAX	1.87	1.62	2.24	1.12	1.71	2.27	1.59	2.40
		MIN	0.93	0.80	0.76	0.37	0.88	1.14	0.79	1.18
		AVG	1.28	1.11	1.39	0.65	1.20	1.81	**1.09**	1.89
		SD	0.26	0.23	0.44	0.21	0.22	0.30	**0.22**	0.33
	fibre	MAX	1.84	1.53	1.86	0.85	1.61	2.28	1.58	2.38
		MIN	0.80	0.66	0.83	0.40	0.70	1.02	0.68	1.07
		AVG	1.35	**1.13**	1.40	0.66	1.19	1.71	1.16	1.79
		SD	0.38	**0.32**	0.39	0.18	0.34	0.47	0.33	0.49

## Data Availability

No new data were created or analyzed in this study.

## Abbreviations

The following notation is used in this article:

$\rho_{f}$	FRP reinforcement ratio
a	shear span
$a_{g}$	maximum aggregate size
b	width of cross-section
d	distance from extreme compression fibre to centroid of tension reinforcement
$f_{c}$	compressive strength of concrete
$f_{ct}$	tensile strength of concrete
h	height of cross-section
$l_{t}$	span length
$E_{c}$	modulus of elasticity of concrete
$E_{f}$	modulus of elasticity of FRP
$E_{s}$	modulus of elasticity of steel
M	bending moment due to the applied load
V	shear force due to the applied load
